# Optimised genetic algorithm-extreme learning machine approach for automatic COVID-19 detection

**DOI:** 10.1371/journal.pone.0242899

**Published:** 2020-12-15

**Authors:** Musatafa Abbas Abbood Albadr, Sabrina Tiun, Masri Ayob, Fahad Taha AL-Dhief, Khairuddin Omar, Faizal Amri Hamzah

**Affiliations:** 1 CAIT, Faculty of Information Science and Technology, Universiti Kebangsaan Malaysia, Bangi, Selangor, Malaysia; 2 Department of Communication Engineering, School of Electrical Engineering, Universiti Teknologi Malaysia, UTM Johor Bahru, Johor, Malaysia; 3 Department of Emergency Medicine, Hospital Canselor Tuanku Muhriz, Universiti Kebangsaan Malaysia Medical Centre, Bandar Tun Razak, Cheras, Kuala Lumpur, Malaysia; University of Engineering & Technology, Taxila, PAKISTAN

## Abstract

The coronavirus disease (COVID-19), is an ongoing global pandemic caused by severe acute respiratory syndrome. Chest Computed Tomography (CT) is an effective method for detecting lung illnesses, including COVID-19. However, the CT scan is expensive and time-consuming. Therefore, this work focus on detecting COVID-19 using chest X-ray images because it is widely available, faster, and cheaper than CT scan. Many machine learning approaches such as Deep Learning, Neural Network, and Support Vector Machine; have used X-ray for detecting the COVID-19. Although the performance of those approaches is acceptable in terms of accuracy, however, they require high computational time and more memory space. Therefore, this work employs an Optimised Genetic Algorithm-Extreme Learning Machine (OGA-ELM) with three selection criteria (i.e., random, K-tournament, and roulette wheel) to detect COVID-19 using X-ray images. The most crucial strength factors of the Extreme Learning Machine (ELM) are: (i) high capability of the ELM in avoiding overfitting; (ii) its usability on binary and multi-type classifiers; and (iii) ELM could work as a kernel-based support vector machine with a structure of a neural network. These advantages make the ELM efficient in achieving an excellent learning performance. ELMs have successfully been applied in many domains, including medical domains such as breast cancer detection, pathological brain detection, and ductal carcinoma in situ detection, but not yet tested on detecting COVID-19. Hence, this work aims to identify the effectiveness of employing OGA-ELM in detecting COVID-19 using chest X-ray images. In order to reduce the dimensionality of a histogram oriented gradient features, we use principal component analysis. The performance of OGA-ELM is evaluated on a benchmark dataset containing 188 chest X-ray images with two classes: a healthy and a COVID-19 infected. The experimental result shows that the OGA-ELM achieves 100.00% accuracy with fast computation time. This demonstrates that OGA-ELM is an efficient method for COVID-19 detecting using chest X-ray images.

## 1. Introduction

Since early December 2019, the Coronavirus disease-2019 (COVID-19) had caused panic around the world. The fast escalation of COVID-19 has resulted in over twenty six millions of infections and approaching nine hundred thousand deaths globally. To date, this pandemic remains a significant challenge because it threatens human life and disrupts the economies of many countries [1, 2].

At present, the detection of viral nucleic acid utilizing real-time reverse transcriptase polymerase chain reaction (RT-PCR) is used as the standard diagnostic method. However, many hyperendemic areas or countries cannot conduct sufficient testing of RT-PCR for tens of thousands of suspected COVID-19 patients. Many efforts have been exerted to detect COVID-19 using computed tomography (CT) images for addressing the lack of reagents such as [3–5]. For example [4], conducted a chest CT for COVID-19 testing with 51 patients and achieved a high sensitivity of 98%. At the same time [5], used the technique of deep learning to detect COVID-19 utilizing CT images. Although employing CT images are useful to detect COVID-19; however, it consumes more time than X-ray imaging. The quality and quantity of CT scanners in several undeveloped regions may be low/limited, thereby leading to an inappropriate detection of COVID-19. X-ray is a well-known and broadly available technique used in diagnostic imaging and plays a vital role in epidemiological studies and clinical care [3, 6]. Numerous ambulatory care facilities have deployed X-ray imaging units (especially in rural regions) for diagnostic imaging. X-ray imaging in real-time significantly accelerates disease detection.

Given these advantages of X-ray imaging, many researchers have exerted efforts to find an accurate COVID-19 detection tool using chest X-ray images [7–9]. Researchers in [10] used artificial intelligence (AI) techniques in the early detection of COVID19 using chest X-ray images. These images were classified using several machine learning algorithms, such as support vector machine (SVM), convolutional neural network (CNN), and random forest (RF). They analyse the performance of SVM, CNN, and RF; and identified that the performance of CNN is the best among the other methods with an accuracy of 95.2% [11], used a deep learning technique for COVID-19 detection based on X-ray images. Their model consisted of three components: anomaly detection head, classification head, and backbone network. The experimental results showed that the model achieves 96.00% sensitivity. While [7], employed CNN for automatic COVID-19 detection tested on X-ray image dataset consisted of patients with COVID-19 and common pneumonia, and healthy persons to automatically detect COVID-19. They obtained 97.82% of accuracy for COVID-19 detection. In [9], the deep features of CNN were extracted and fed to the SVM for COVID-19 detection. The X-ray image datasets were collected from Open-I repository, Kaggle, and GitHub. The results showed that the accuracy of SVM and 50 layer Residual Network (ResNet50) reaches 95.38%. While the authors in [12] presented a ResNet model in their work where they considered data imbalance as one of the primary concerns. They have used 70 COVID-19 patients. The evaluation result showed 96% sensitivity, 70.7% specificity for ResNet. The work in [13] has experimented on a dataset combination of 70 COVID-19 images from one source [14] and non-COVID-19 images from Kaggle chest X-ray dataset. They proposed the Bayesian CNN model, which improves the detection rate from 85.7% to 92.9% along with the VGG16 model [15]. Further, in [16] the authors have presented a COVID-19 diagnosis system using a variant of CNN named Resnet50. The system is used 89 samples for COVID-19 infected, and 93 samples for healthy participants. The collected dataset was split into two sets like training and testing in a proportion of 80%, and 20%. The diagnosis process obtained 98.18% accuracy. In [17] the authors have developed an automated COVID-19 diagnosis system using several pre-trained models with a small number of X-ray images. From the experimental results, it was shown that NASNetLarge performed comparatively better and achieved 98% accuracy.

On the other hand, some researchers preferred to use Extreme Learning Machine (ELM) because of its superiority over conventional SVMs [18–20] in terms of 1) its ability to prevent overfitting, 2) its usability on binary and multi-type classifiers, and 3) its kernel-based ability similar to SVM when working with a NN structure. These advantages make ELM efficient in achieving a better learning performance [18].

The distinct features of ELM, including its good generalisation, rapid training, and universal approximation/classification capability, has rendered it to be highly prominent in the AI and machine learning [21]. ELM is more suitable for single hidden layer feedforward neural networks (SLFNs) because of its excellent learning accuracy/speed, as proven in many applications [22]. ELM has better and faster generalisation performance than SVM and backpropagation-based NNs [21, 23, 24]. Besides, the effectiveness of the ELM has been proven in several medical domains such as ductal carcinoma in situ detection [25] and pathological brain detection [26, 27]. In order to further enhance the ELM [28], optimised the input-hidden layer weight and bias using Optimised Genetic Algorithm and named it as Optimised Genetic Algorithm-Extreme Learning Machine (OGA-ELM). The OGA-ELM was tested on spoken language identification and showed an excellent performance compared to ELM. However, to the best of our knowledge, no research has used ELM classifiers for detecting COVID-19 based on chest X-ray images.

Although the performance of those works was acceptable, more enhancement still needs to be done in terms of accuracy, features dimension, memory space, and computational time. The required memory space and the computational time are affected by the dimensionality of the features (number of features). The higher dimensionality requires a long computational time and large memory space [29–31]. In order to address these issues, some works have used dimensionality reduction and parallel processing techniques. Therefore, this work aims to the following contributions:

- Adapt the principal component analysis (PCA) to reduce the histogram of oriented gradients (HOG) features.
- Improve the accuracy by employing the OGA-ELM classifier to classify the chest X-ray images into healthy and COVID-19 infected.
- Evaluate the OGA-ELM performance with three selection criteria (i.e., random, K-tournament, and roulette wheel) for COVID-19 detection based on X-ray images.
- Evaluate the proposed COVID-19 detection system in terms of effectiveness and efficiency.

HOG is one of the most popular feature extraction approaches that has widely used in various image processing domains, including medical domains [32–34]. PCA is one of the most well-known schemes for dimensionality reduction [35]. This approach condenses most of the information in a dataset into a small number of dimensions.

The organisation of the paper is as follows: The proposed method (COVID-19 detection system) is provided in Section 2. Section 3 deliberates the conducted experiments and their findings. Section 4 provides general conclusions and suggestions for future research.

## 2. Method

### 2.1. General overview

**Fig 1:**
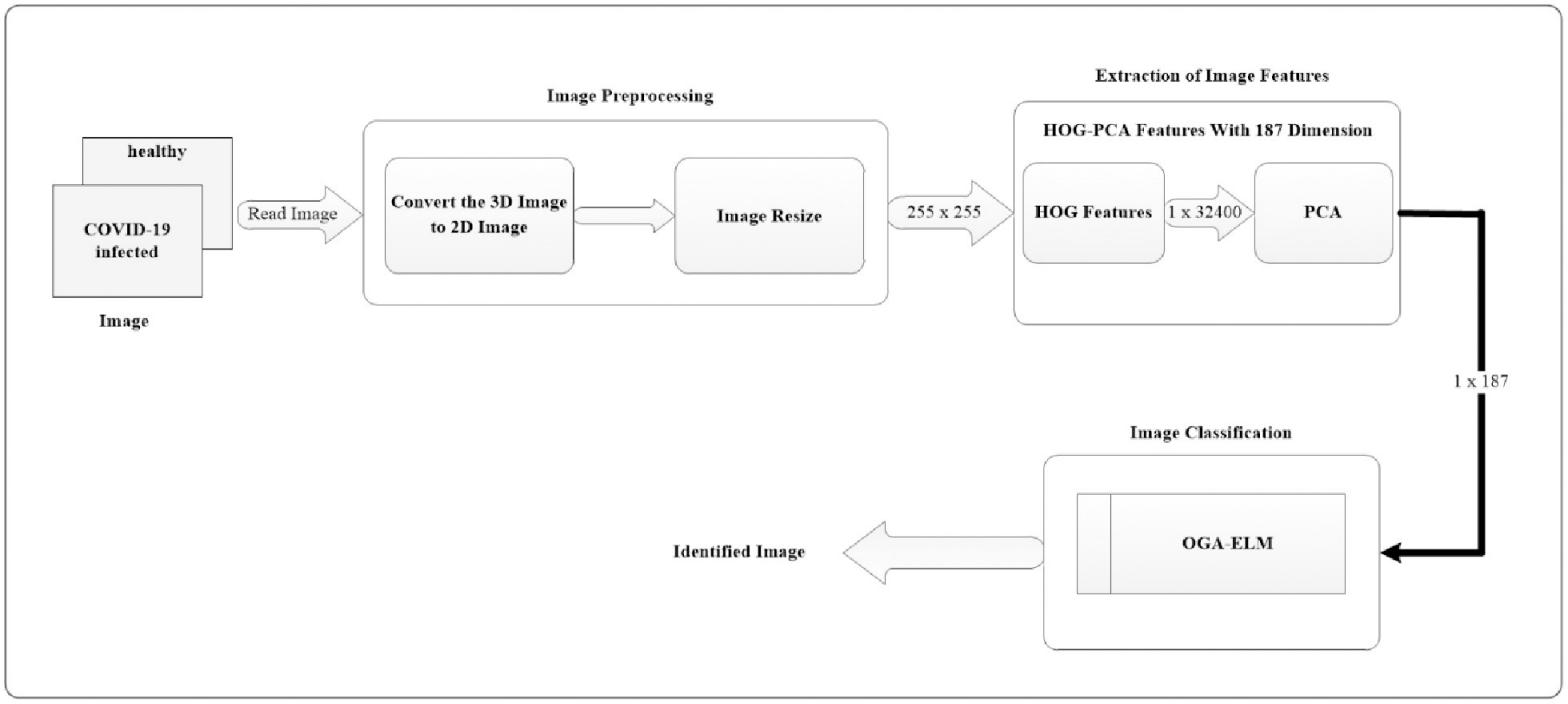
Illustrative block diagram of the proposed COVID-19 detection system.

The overall overview of the proposed COVID-19 detection system using the OGA–ELM approach is shown in Fig 1. The diagram illustrates various processing blocks used to create the COVID-19 detection system on chest X-ray images. The following subsections will discuss each of the processing blocks, as shown in the COVID-19 detection system (Fig 1).

### 2.2. Image preprocessing

The preprocessing of images consisting of two steps: image conversion and resize. The first step is to read the image and check its dimensionality. A 3D image must be converted to a 2D image. Secondly, we resize the dimensionality of the 2D image to (255 × 255). The output of this stage will be used as the input for extracting the features of the image.

### 2.3. Extraction of image features

**Fig 2:**
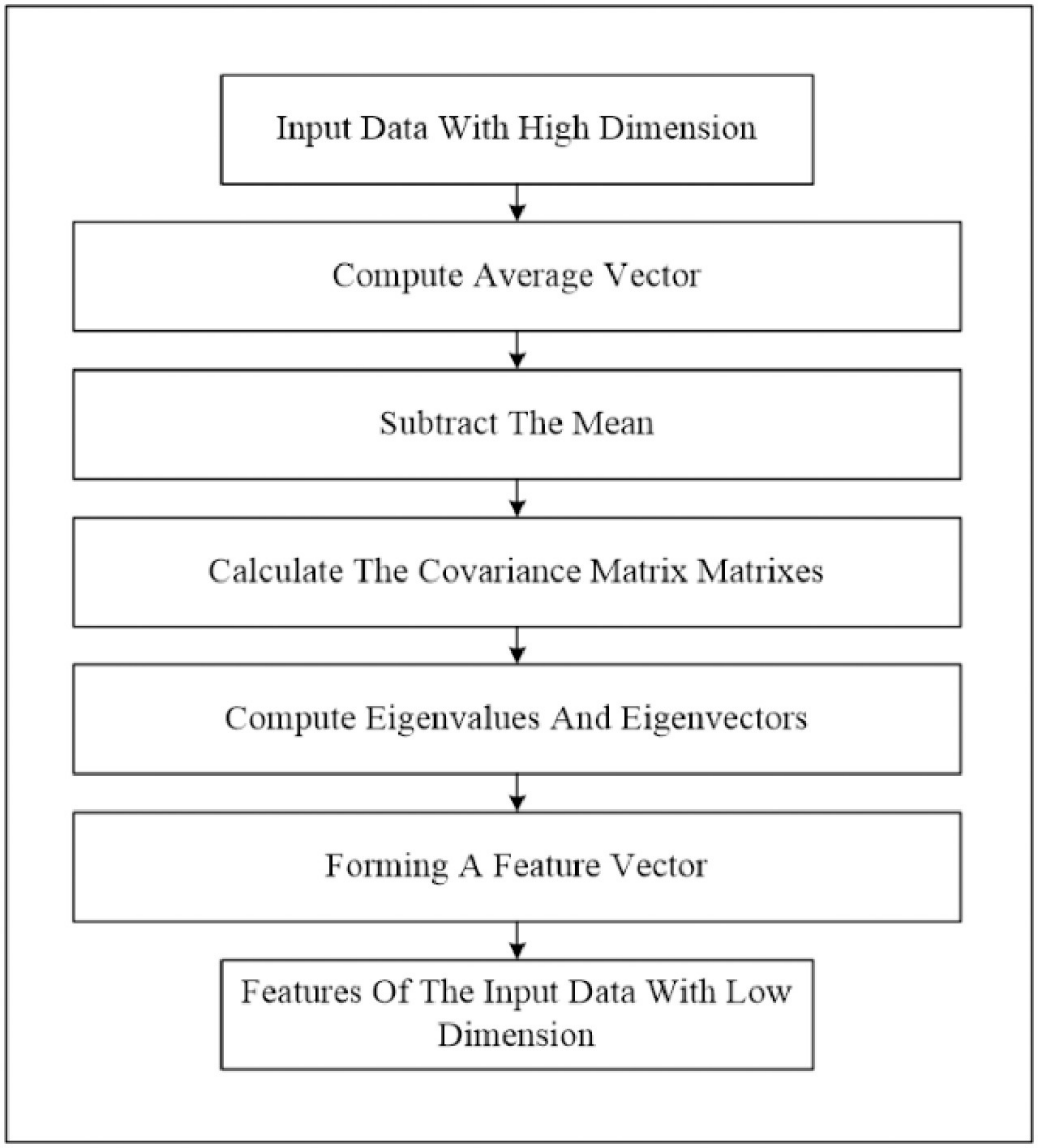
PCA steps.

**Fig 3:**
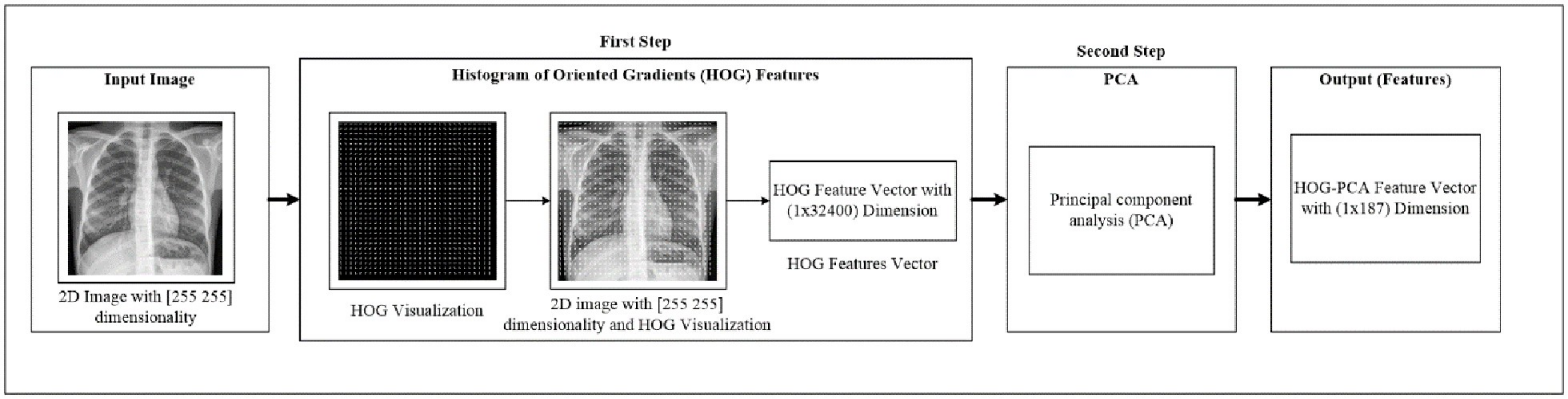
Feature extraction steps.

At this stage, we perform two phases. Firstly, we extract the image feature using the histogram of oriented gradients (HOG) feature extraction method. HOG is a popular feature used in many image processing applications [36–38]. The HOG can be performed by dividing the image into small parts that are named cells. Each cell compiles a histogram of gradient direction for the pixel within the cell. The HOG method has four steps to extract features. The first step is calculating the gradient values to obtain the point of discrete derivative mask in the horizontal and vertical direction. The second step is the spatial orientation binning. This step has a function to give a result of a cell histogram by a voting process. Each pixel of the image within the casts a weighted vote for orientation in accordance with the closest bin in the range 0 to 180 degrees. In the third step, there is the HOG descriptor to normalize cell and histogram from the entire block region to be a vector form. The fourth step is performed by applying the block normalization. The output of the HOG feature extraction approach is a vector with a dimension of (1 × 32,400) per image and (188 × 32,400) for the entire dataset. The second phase is to apply the principal component analysis (PCA) dimensionality reduction on HOG features. PCA method has used mostly as pattern recognition system because it is very useful as the data reducing technique. The PCA processing steps can be seen in Fig 2. This step reduces the high dimensionality of the HOG features from (188 × 32,400) to (188 × 187) for the entire dataset. It aims to overcome the time consumption and limited resources (requiring a large memory). The final output of feature extraction is the HOG–PCA features with (188 × 187) dimensionality for the entire dataset that will be used as input in the classification step. Fig 3 depicts the feature extraction steps in detail.

### 2.4. Image classification: OGA-ELM

We adopt the OGA–ELM from [28] to classify the chest X-ray image dataset into healthy and COVID-19 infected. It utilises three selection criteria, where the input values (the weight and bias) of the hidden nodes are tuned by utilizing mutation, crossover, and selection operations. The parameters of the OGA and ELM used in the experiments are summarised in Table 1.

**Table 1:** Parameters of the ELM and OGA [28].

ELM		OGA	
Parameter	Value	Parameter	Value
C	Combined bias and input weight	Number of iterations	100
*ρ*	Output weight matrix	Population size	50
Input weight	−1 to 1	Crossover	Arithmetical
Value of the biases	0–1	Mutation	Uniform
Input node numbers	Input attributes	Population of the crossover (POPC)	Refers to the crossover population, which is 70% of the population.
Hidden node numbers	(100–300), with step or increment of 25	Population of the mutation (POPM)	Refers to the mutation population, which is 30% of the population.
Output neuron	Class value	Gamma value	0.4
Activation function	Sigmoid	Tournament size	3

*N* is a collection of featured samples (X_i_, t_i_), where X_i_ = [x_i1_, x_i2_, …, x_in_]^T^ ∈ R^n^, and t_i_ = [t_i1_, t_i2_, …, t_im_]^T^ ∈ R^m^.

Where:

X_i_ is the input which is extracted features from HOG-PCA;

t_i_ is the true values (expected output).

At the beginning of OGA–ELM, the values of input weights, and the thresholds of hidden nodes are randomly defined and characterised as chromosomes.

C={w11,w12,…,w1n,w21,w22,…,w2n,wL1,wL2,…,wLn,b1,…,bL}

Where:

w_ij_: refers to the weight value that relates the *ith* hidden node and the *jth* input node, w_ij_∈ [-1, 1];

b_i_: refers to *ith* hidden node bias, b_i_∈ [0, 1];

n: refers to the number of input node; and

L: refers to the number of hidden node.

(1+n) × L represents the chromosome dimensionality, that is, the (1+n) × L parameters that need to be optimised.

The fitness function of OGA–ELM is calculated, as shown in Eq (1) [22] to maximise the accuracy.

$$f(C)=\sqrt{\frac{\sum_{j}^{N}||\sum_{k}^{L}\rho_{k}g(w_{k}x_{j}+\;b_{k})-t_{j}{\left|\right|}_{2}^{2}}{N}}$$ (1)

Where:

ρ = matrix of the output weight;

t_j_ = expected output; and

N = training samples number.

Then,

Hρ=T (2)

Where *T* is the expected output.

$$H={\left[\begin{array}{ccc} g(w_{1}.X_{1}+b_{1}) & \cdots & g(w_{L}.X_{1}+b_{L})\\ \vdots & \ldots & \vdots \\ g(w_{1}.X_{N}+b_{1}) & \cdots & g(w_{L}.X_{N}+b_{L}) \end{array}\right]}_{N\times L}$$ (3)

$$\rho ={\left[\begin{array}{c} \rho_{1}{}^{T}\\ \vdots \\ \rho_{L}{}^{T} \end{array}\right]}_{L\times m}\text{and}\;T={\left[\begin{array}{c} t_{1}{}^{T}\\ \vdots \\ t_{N}{}^{T} \end{array}\right]}_{N\times m}$$

In [20], H indicates the NN hidden layer output matrix, and the *ith* column in *H* indicates the *ith* hidden layer nodes on the input nodes. Activation function *g* is infinitely distinguishable when the desired number of hidden nodes is *L ≤ N*. The output weights ρ can be specified by discovering the least-squares solution, as shown in the following equation:

$$\rho =H^{†}T,$$ (4)

where *H*^†^ refers to the Moore–Penrose generalised inverse of *H*. Thus, the weights of output (*ρ*) are calculated through a mathematical transformation that avoids any long training phrase where the network parameters are iteratively tuned with several suitable learning parameters (e.g., iterations and learning rate).

***First***, generate the initial population (P) randomly, p = {C_1_, C_2_…C_50_}.

***Second***, calculate the fitness value for each chromosome (C) of the population using Eq (1).

***Third***, the chromosomes are arranged based on their fitness values *f(C)*. Next, we select a pair of parents from the present population for the operation of crossover to create a pair of new children to the new population. One of the three different selection criteria will be used: random, K-tournament, and roulette wheel.

Random selection criterion refers to the process that randomly picks a chromosome from the population to be used in one of the two operations: crossover or mutation. In the random selection criterion, every single chromosome of the population has an equal chance of being chosen.

K-tournament selection criterion chooses a number of solutions (tournament size) randomly and then selects the best of the chosen solutions to be as a parent.

In the roulette wheel selection criterion, the circular wheel is separated into population size (PS) pies, where PS is the number of individuals (chromosomes) in the population. Each chromosome attains a share of the circle proportionate to its fitness value. As shown on the wheel of circumference, a selection point is picked by which the wheel is rotated. The area of the wheel landing in front of the selection point is picked as the parent. The same process is repeated for selecting the second parent. Obviously, the fitter chromosome attains a larger pie in the wheel and thus a larger chance of stopping in front of the selection point. Hence, the possibility for a chromosome to be selected is directly determined by its fitness.

***Fourth***, the arithmetic crossover is applied to exchange information between the two previously selected parents. The new children obtained by crossover operations are saved into the Population of the Crossover (POPC) until it reaches 70% of the population. The explanation of the arithmetic crossover is represented by the following formulae:

$${\text{Child}}_{1}=\alpha .\text{x}\;+\;(1-\alpha ).\text{y}$$ (5)

$${\text{Child}}_{2}=\alpha .\text{y}\;+\;(1-\alpha ).\text{x}$$ (6)

Subject to the boundaries (upper bounds and lower bounds for the input-hidden layer weights [-1, 1], while for the hidden layer biases [0, 1]). In case the value of the gene has gone beyond the max (upper bound), then we make it equal to the max (upper bound). While in case the value of the gene has gone lower than the min (lower bound), then we make it equal to the min (lower bound). α is a randomly generated array with the size of the chromosome, and each value of this array is randomly generated in a range of -gamma and gamma+1 which is (-0.4, 1.4). x and y represent the first and second selected parents.

**Fig 4:**
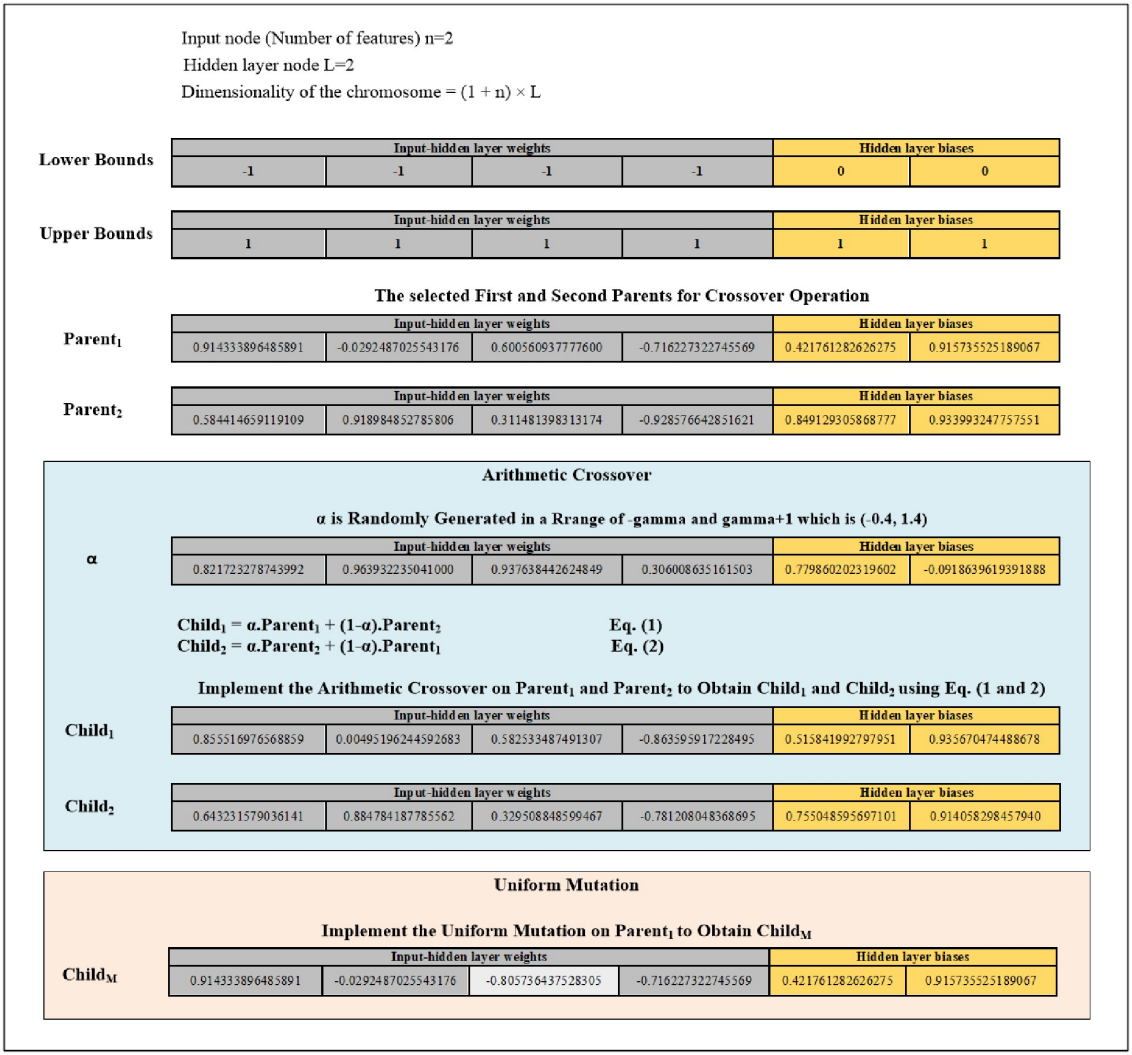
Diagram of the arithmetic crossover and uniform mutation operations example.

***Fifth***, criteria of the random selection are used to randomly choose a chromosome from the present population before implementing mutation. Mutation is applied to alter the chromosome’s genes that are randomly selected. This work utilises uniform mutation. The uniform mutation works to substitute the selected gene’s value with a uniform random value chosen from the gene’s user-specified upper and lower bounds (for the input-hidden layer weights [-1, 1] while for the hidden layer biases [0, 1]). The new child obtained from mutation will be saved into the Population of the Mutation (POPM) until the POPM reaches 30% of the population. Fig 4 provides an example of the arithmetic crossover and uniform mutation operations.

**Fig 5:**
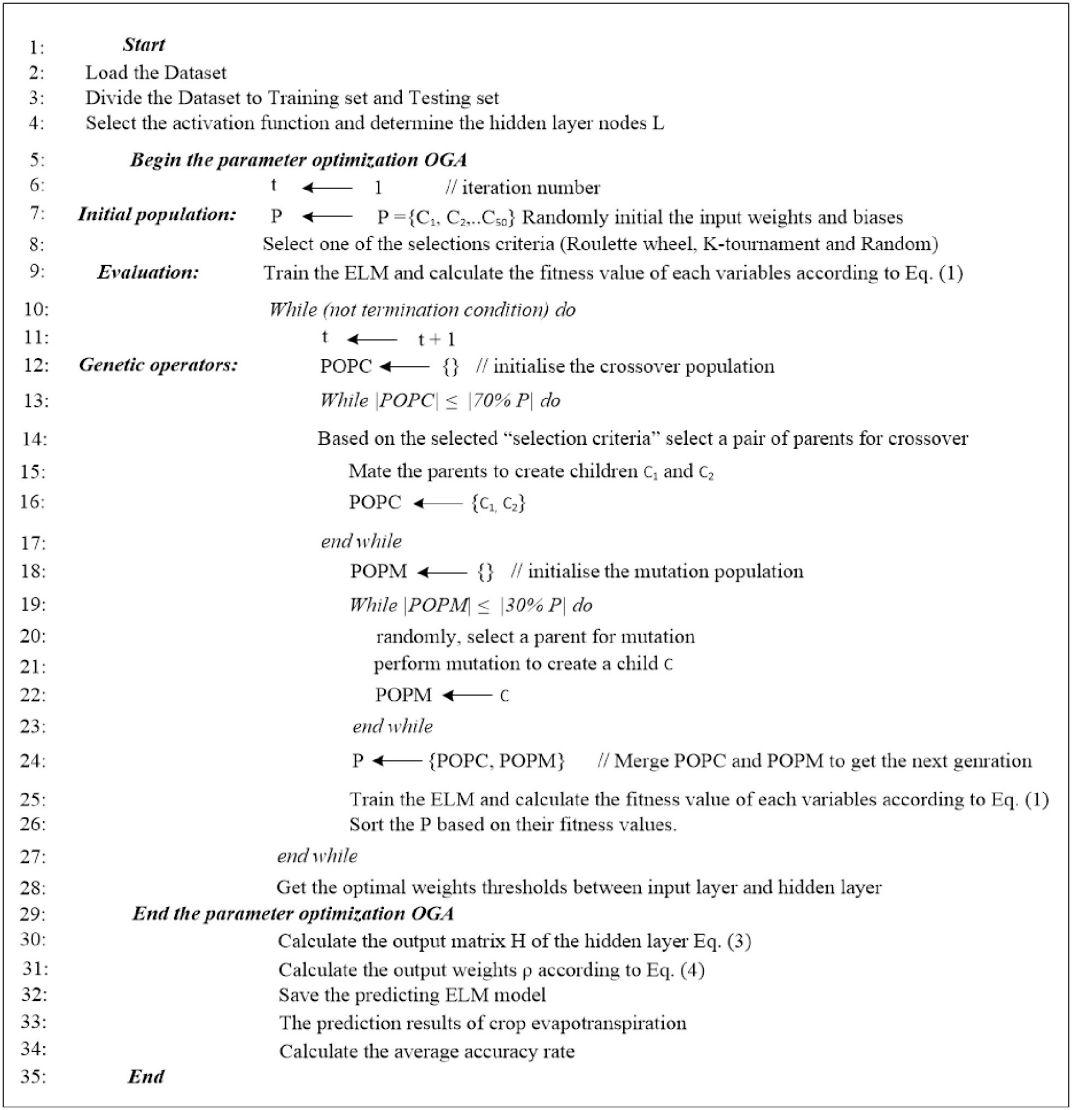
Pseudocode of the OGA-ELM [28].

**Fig 6:**
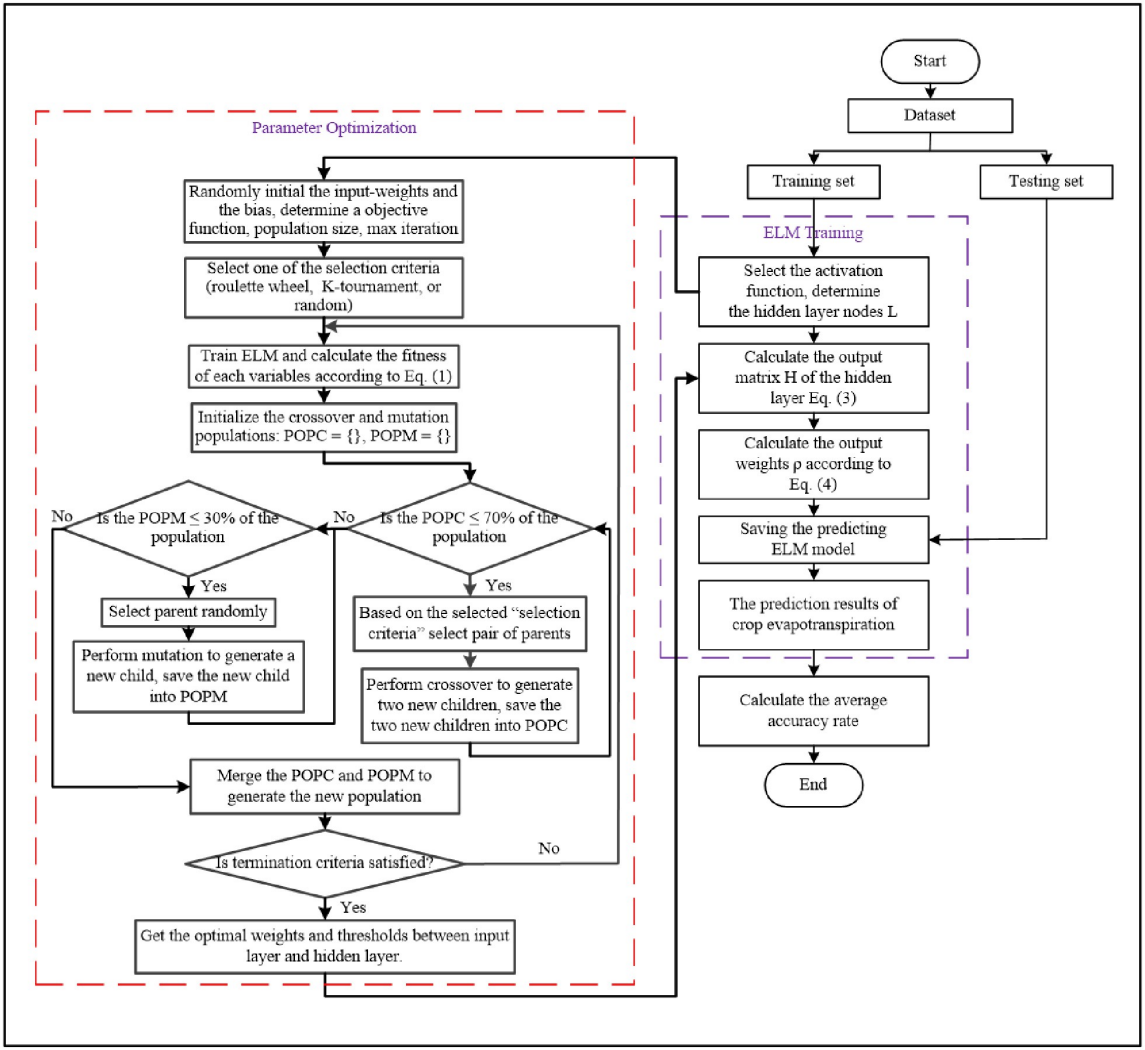
OGA-ELM’s flowchart [28].

After the selection, mutation, and crossover operations are completed, a new population is created via integrating the POPM and POPC. The following iteration will be continued along with this new population, and this process will be repeated. The iterative process could be stopped when either the results have converged or the iteration numbers is exceeded the maximum limit. OGA–ELM’s pseudocode and flowchart are shown in Figs 5 and 6, respectively.

## 3. Experiments and results

### 3.1. Image dataset

**Fig 7:**
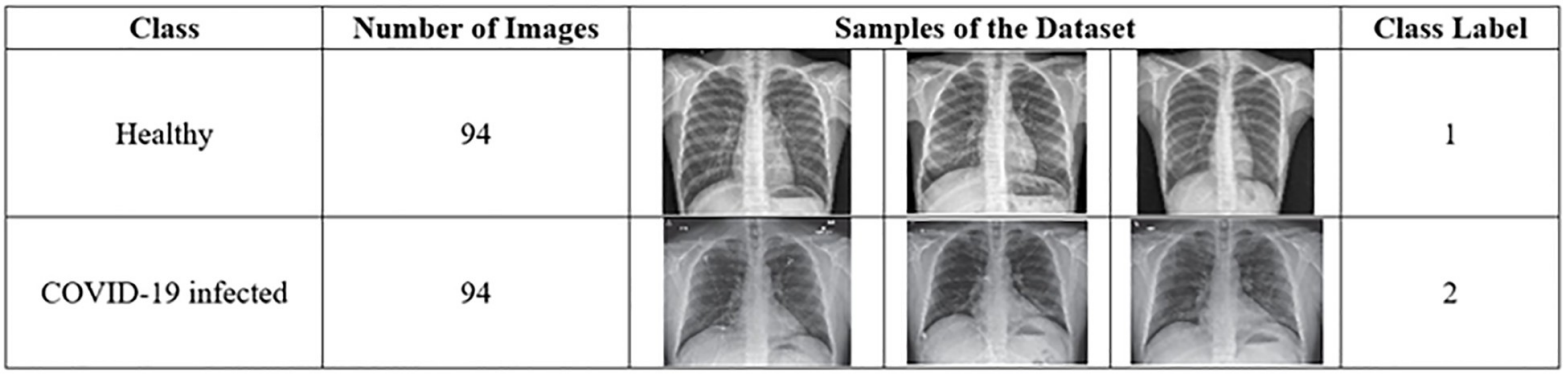
Description of the dataset.

This study used a dataset downloaded from [14] that contains chest X-ray images. The dataset contains two main classes: healthy and COVID-19 infected classes. The healthy class refers to the chest X-ray image of a patient negative for COVID-19 or an uninfected patient. The COVID-19 infected class refers to the X-ray image of a patient positive for COVID-19 or an infected patient. Each class of the dataset contains 94 images, and the total number of images in the entire dataset is 188. In this study, we divided the dataset to 60% for training (i.e. 56 images for each class, total is 112 images), and 40% for testing (i.e. 38 images for each class, total is 76 images). Fig 7 describes the dataset. Table 2 illustrates the dimensionality of feature extraction steps for a single image and for the entire dataset images.

**Table 2:** Feature extraction step dimensionality for single image and entire dataset images.

Feature Extraction	Single Image Dimensionality	All Dataset Dimensionality
First Step: HOG Features	(1 × 32,400)	(188 × 23,400)
Second Step: HOG–PCA Features	(1 × 187)	(188 187)

### 3.2. Results and discussion

OGA-ELM (random, K-tournament, and roulette wheel) underwent several classification experiments based on the formulated dataset by varying the hidden neuron numbers in the range of 100–300 with an increment step of 25. Hence, the total experiment numbers for each approach was 9. Each experiment had 100 iterations. It is worth mentioning that all the experiments have been implemented in MATLAB R2019a programming language over a PC Core i7 of 3.20 GHz with 16 GB RAM and SSD 1 TB (Windows 10).

The evaluation was based on the study in [39], where varying measures were applied. The study was selected because it tackles the issue of classifier evaluation while providing effective measures. The performance of the learning algorithms can be evaluated in several methods using supervised machine learning. A confusion matrix that has records of identified examples of each class in accordance with their correction rate was used to create the classification quality.

Hence, a number of evaluation measures were utilised in the evaluation of the three proposed approaches: OGA–ELM (random, K-tournament, and roulette wheel). The evaluation measures were based on the ground truth that requires applying the model to predict the answer in accordance with the evaluation dataset from the comparison between the actual answer and the predicted target. The measures of the evaluation were used to compare the three proposed approaches: OGA–ELM (random, K-tournament, and roulette wheel) in terms of false negative, true negative, false positive, true positive, recall, accuracy, G-mean, precision, and F-measure. Eqs (7–11) [22, 40] depict the study’s evaluation measures.

$$\text{accuracy}=\frac{tp+tn}{tp+tn+fn+fp}$$ (7)

$$\text{precision}=\frac{tp}{tp+fp}$$ (8)

$$\text{recall}=\frac{tp}{tp+fn}$$ (9)

$$F-\text{measure}=\frac{\left(2\;\times \;precision\;\times \;recall\right)}{\left(precision\;+\;recall\right)}$$ (10)

$$G-\text{Mean}=\sqrt[{2}]{recall\times precision\;}$$ (11)

Where *tn* indicates true negative, *tp* refers to true positive, *fn* indicates false negative, and *fp* refers to false positive.

**Fig 8:**
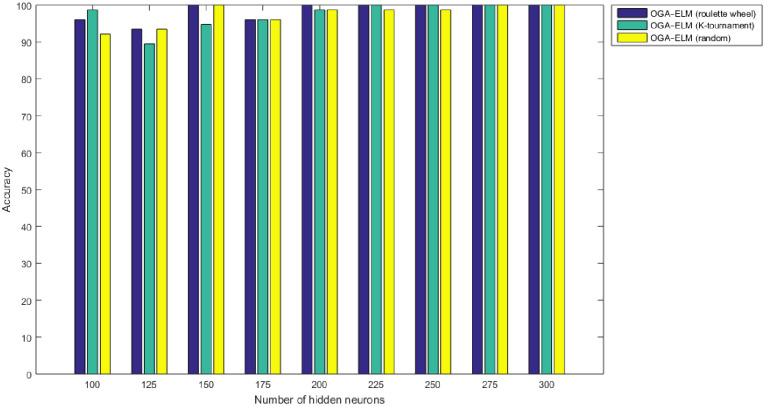
Accuracy results of the OGA–ELM model using random, K-tournament, and roulette wheel.

**Fig 9:**
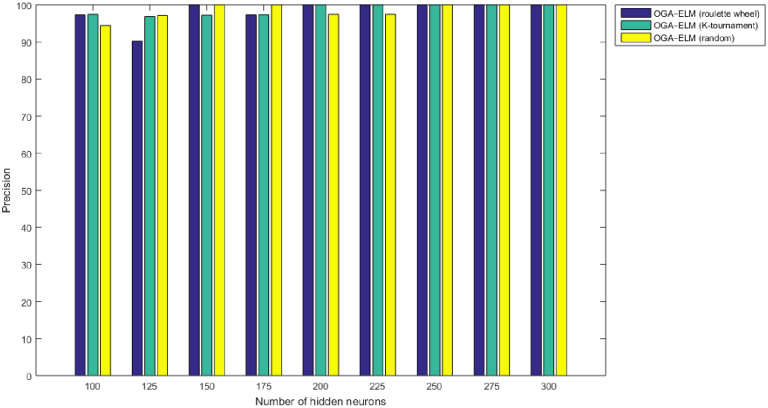
Precision results of the OGA–ELM model using random, K-tournament, and roulette wheel.

**Fig 10:**
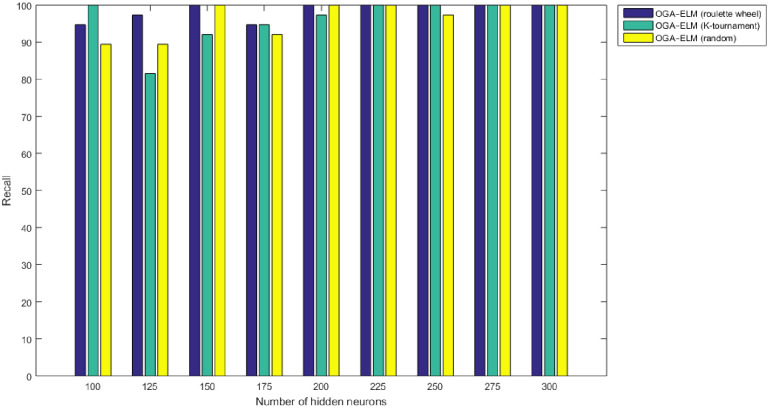
Recall results of the OGA–ELM model using random, K-tournament, and roulette wheel.

**Fig 11:**
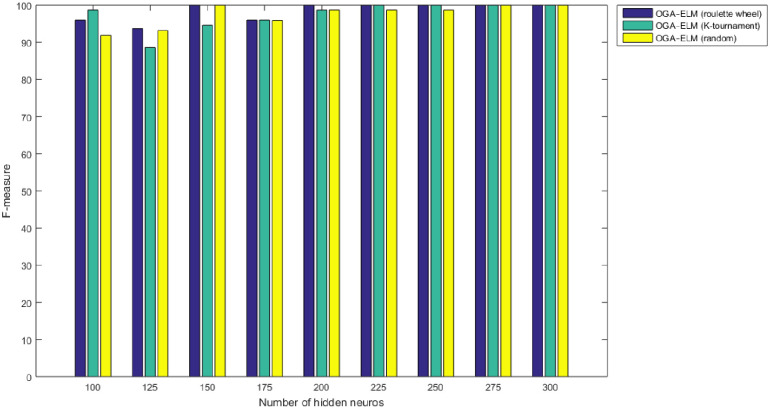
F-measure results of the OGA–ELM model using random, K-tournament, and roulette wheel.

**Fig 12:**
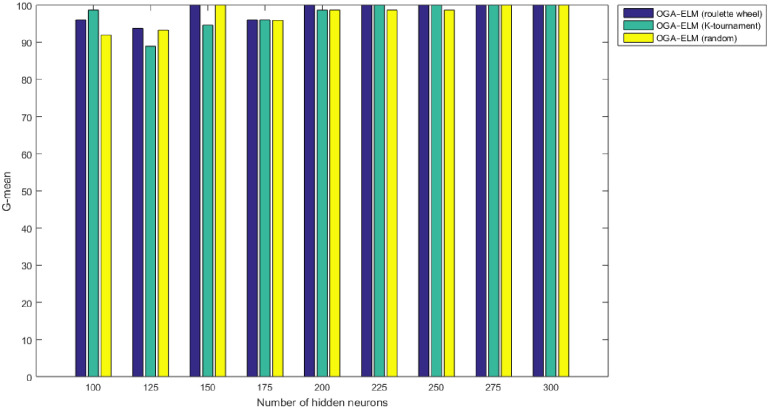
G-mean results of the OGA–ELM model using random, K-tournament, and roulette wheel.

**Fig 13:**
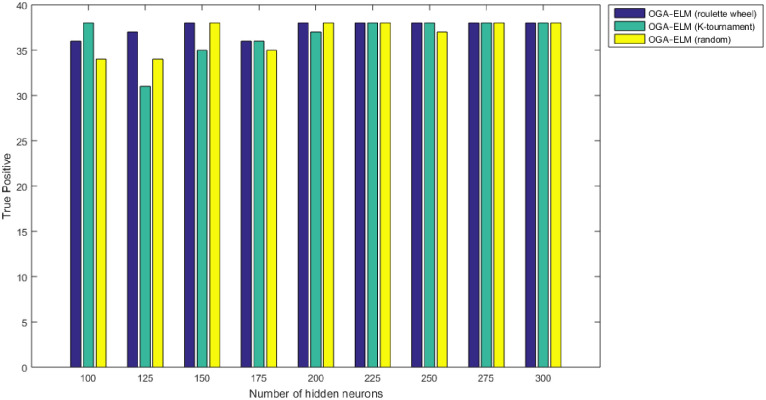
True positive results of the OGA–ELM model using random, K-tournament, and roulette wheel.

**Fig 14:**
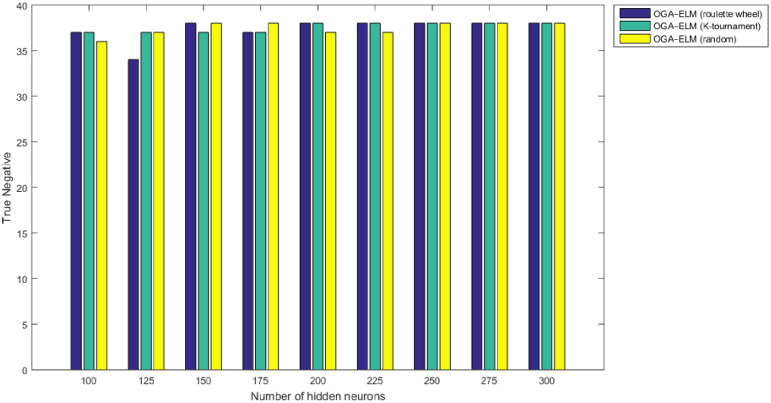
True negative results of the OGA–ELM model using random, K-tournament, and roulette wheel.

**Fig 15:**
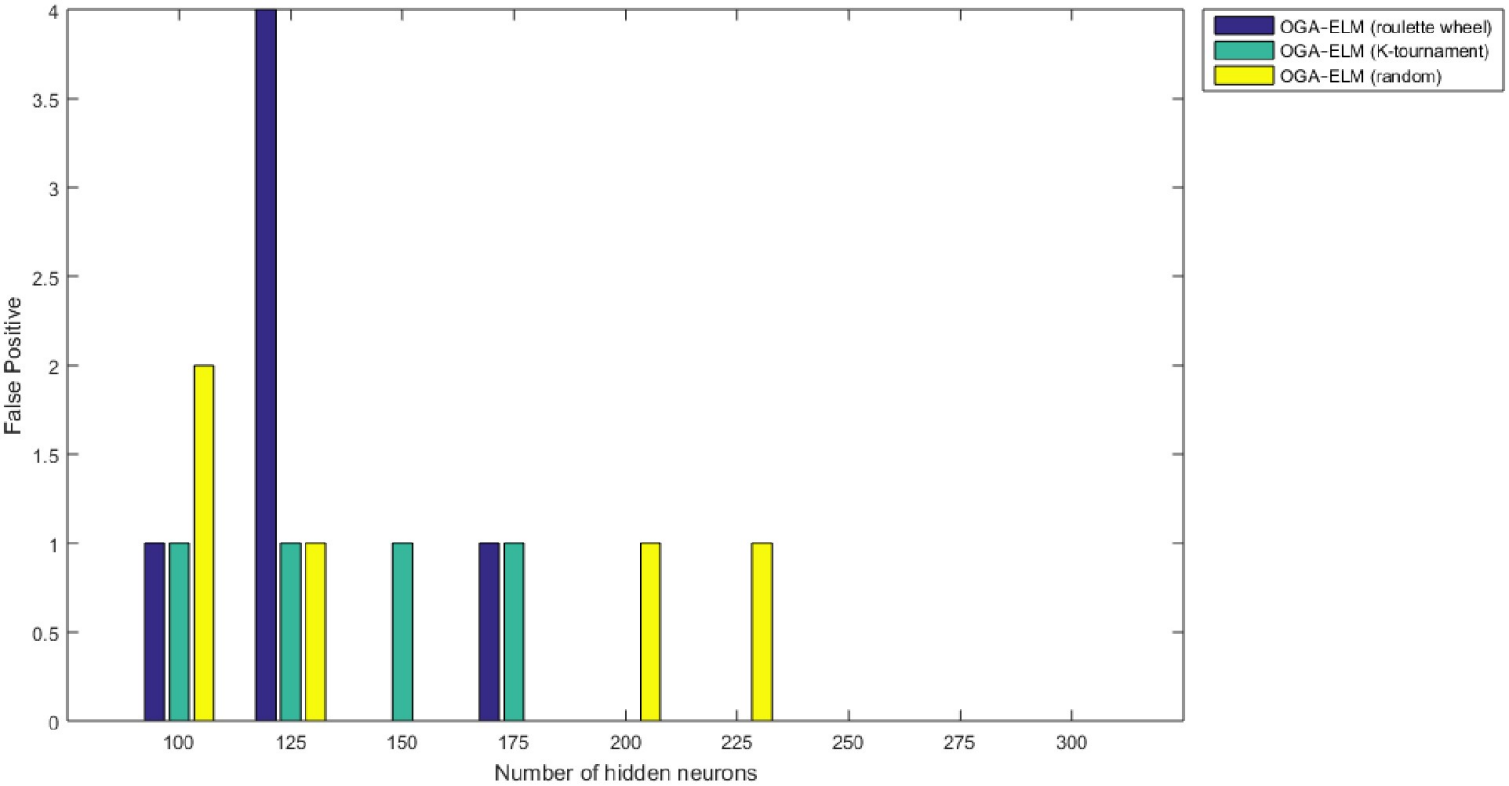
False positive results of the OGA–ELM model using random, K-tournament, and roulette wheel.

**Fig 16:**
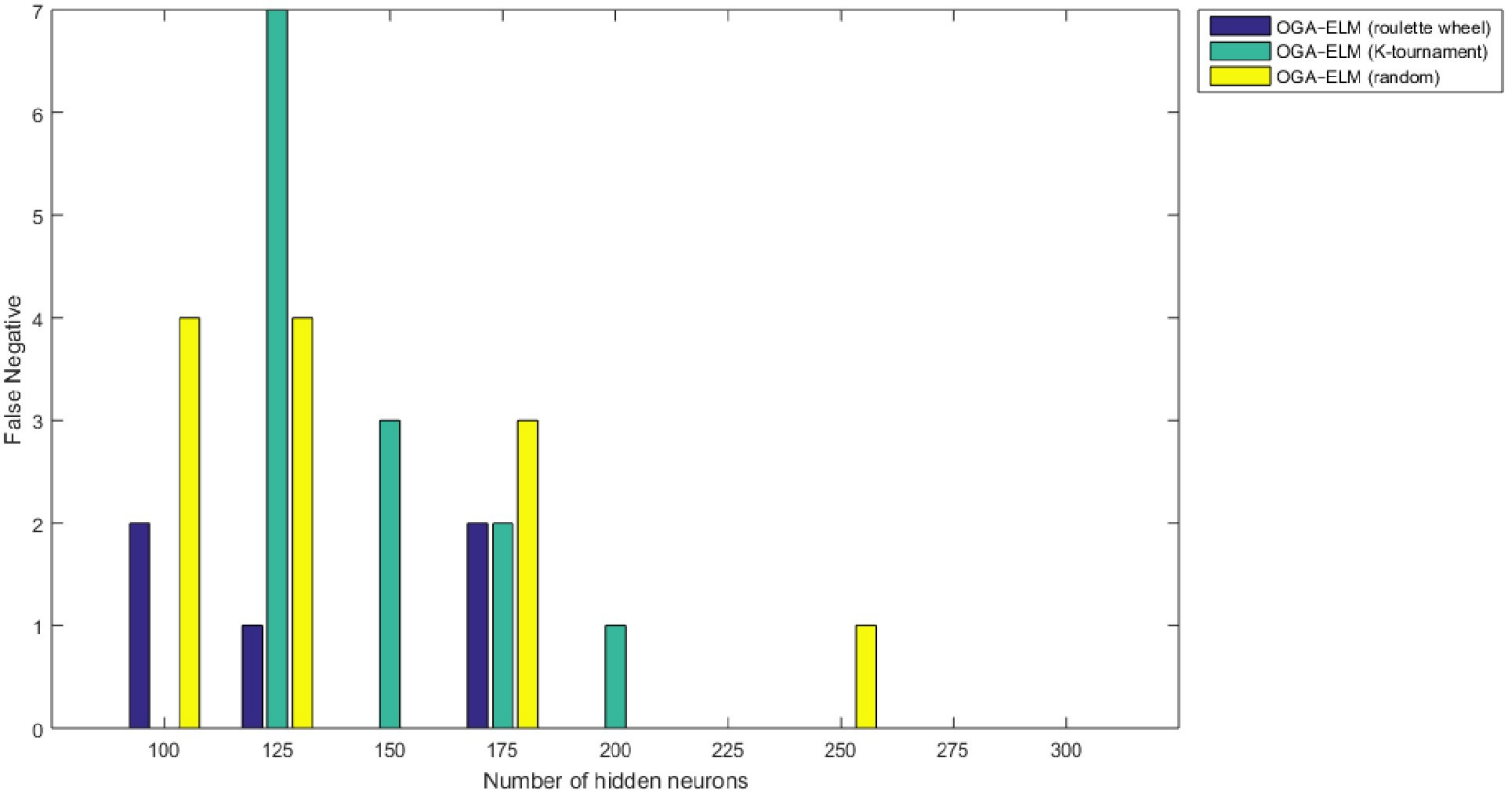
False negative results of the OGA–ELM model using random, K-tournament, and roulette wheel.

**Fig 17:**
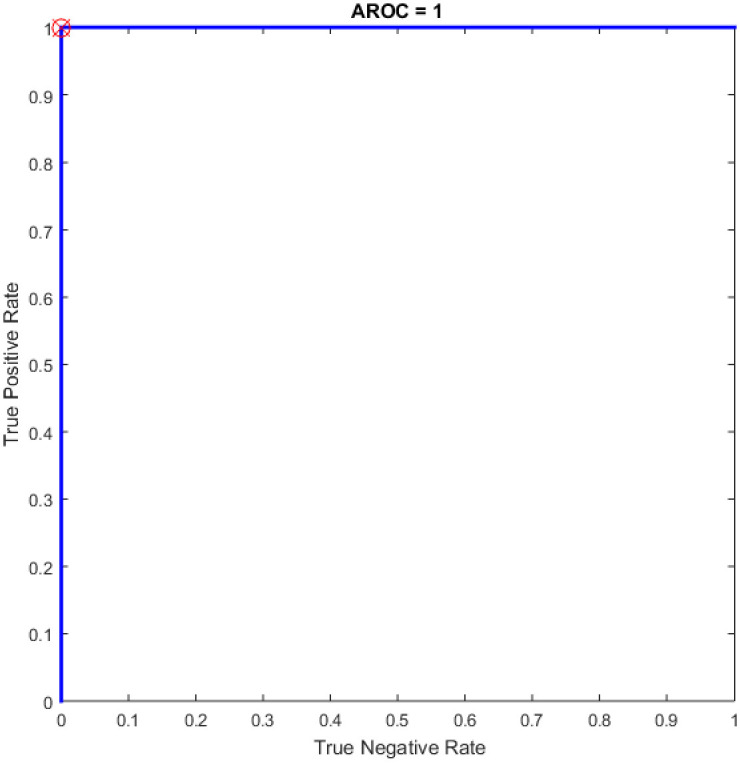
ROC of the OGA–ELM for the highest result.

Figs 8–16 demonstrate the comparative results between the three proposed approaches; OGA–ELM (random, K-tournament, and roulette wheel) in terms of false negative, true negative, false positive, true positive, recall, accuracy, G-mean, precision, and F-measure for all the conducted experiments. An important observation here is that the three approaches achieved the highest accuracy with various numbers of neurons, as shown in Fig 8. The achieved accuracy of the three proposed approaches: OGA–ELM (random, K-tournament, and roulette wheel) was 100.00% for OGA–ELM (K-tournament) with 225–300 neurons; OGA–ELM (roulette wheel) with 150, 200–300 neurons; and OGA–ELM (random) with 150, 275, and 300 neurons. Tables 3–5 present the evaluation measures results of the OGA–ELM (random, K-tournament, and roulette wheel) through all the experiments. Furthermore, Fig 17 shows Receiver Operating Characteristic (ROC) analysis of the proposed OGA-ELM for the highest results.

**Table 3:** Evaluation results based on OGA–ELM (roulette wheel) model.

Number of Hidden Neurons	tp	tn	fp	fn	Accuracy	Precision	Recall	F-measure	G mean	Computational Training/Testing Time in Second
**100**	36	37	1	2	96.05	97.30	94.74	96.00	96.01	35.0106
**125**	37	34	4	1	93.42	90.24	97.37	93.67	93.74	40.2791
**150**	38	38	0	0	100.00	100.00	100.00	100.00	100.00	35.6772
**175**	36	37	1	2	96.05	97.30	94.74	96.00	96.01	40.1244
**200**	38	38	0	0	100.00	100.00	100.00	100.00	100.00	43.3277
**225**	38	38	0	0	100.00	100.00	100.00	100.00	100.00	37.9042
**250**	38	38	0	0	100.00	100.00	100.00	100.00	100.00	40.5361
**275**	38	38	0	0	100.00	100.00	100.00	100.00	100.00	48.7479
**300**	38	38	0	0	100.00	100.00	100.00	100.00	100.00	40.7242

**Notes**: where tn indicates true negative, tp refers to true positive, fn indicates false negative, and fp refers to false positive.

**Table 4:** Evaluation results based on OGA–ELM (K-tournament) model.

Number of Hidden Neurons	tp	tn	fp	fn	Accuracy	Precision	Recall	F-measure	G mean	Computational Training/Testing Time in Second
**100**	38	37	1	0	98.68	97.44	100.00	98.70	98.71	31.4285
**125**	31	37	1	7	89.47	96.88	81.58	88.57	88.90	32.4359
**150**	35	37	1	3	94.74	97.22	92.11	94.59	94.63	32.0790
**175**	36	37	1	2	96.05	97.30	94.74	96.00	96.01	33.4369
**200**	37	38	0	1	98.68	100.00	97.37	98.67	98.68	36.0247
**225**	38	38	0	0	100.00	100.00	100.00	100.00	100.00	35.3846
**250**	38	38	0	0	100.00	100.00	100.00	100.00	100.00	36.1353
**275**	38	38	0	0	100.00	100.00	100.00	100.00	100.00	35.8372
**300**	38	38	0	0	100.00	100.00	100.00	100.00	100.00	37.1120

**Table 5:** Evaluation results based on OGA–ELM (random) model.

Number of Hidden Neurons	tp	tn	fp	fn	Accuracy	Precision	Recall	F-measure	G mean	Computational Training/Testing Time in Second
**100**	34	36	2	4	92.11	94.44	89.47	91.89	91.93	28.4201
**125**	34	37	1	4	93.42	97.14	89.47	93.15	93.23	30.2151
**150**	38	38	0	0	100.00	100.00	100.00	100.00	100.00	31.4233
**175**	35	38	0	3	96.05	100.00	92.11	95.89	95.89	33.0367
**200**	38	37	1	0	98.68	97.44	100.00	98.70	98.71	33.9093
**225**	38	37	1	0	98.68	97.44	100.00	98.70	98.71	34.6111
**250**	37	38	0	1	98.68	100.00	97.37	98.67	98.68	35.3741
**275**	38	38	0	0	100.00	100.00	100.00	100.00	100.00	36.5370
**300**	38	38	0	0	100.00	100.00	100.00	100.00	100.00	36.1408

A crucial observation can be concluded on the basis of the experimental results in Tables 3–5 and Figs 8–16. The OGA with three criterion selection, namely, random, K-tournament, and roulette wheel can generate appropriate biases and weights for the single hidden layer of the ELM to reduce classification errors. Avoiding inappropriate biases and weights prevents the ELM to be stuck in the local maxima of biases and weights. Therefore, the performance of OGA–ELM (random, K-tournament, and roulette wheel) is impressive, with an accuracy of 100.00%.

**Fig 18:**
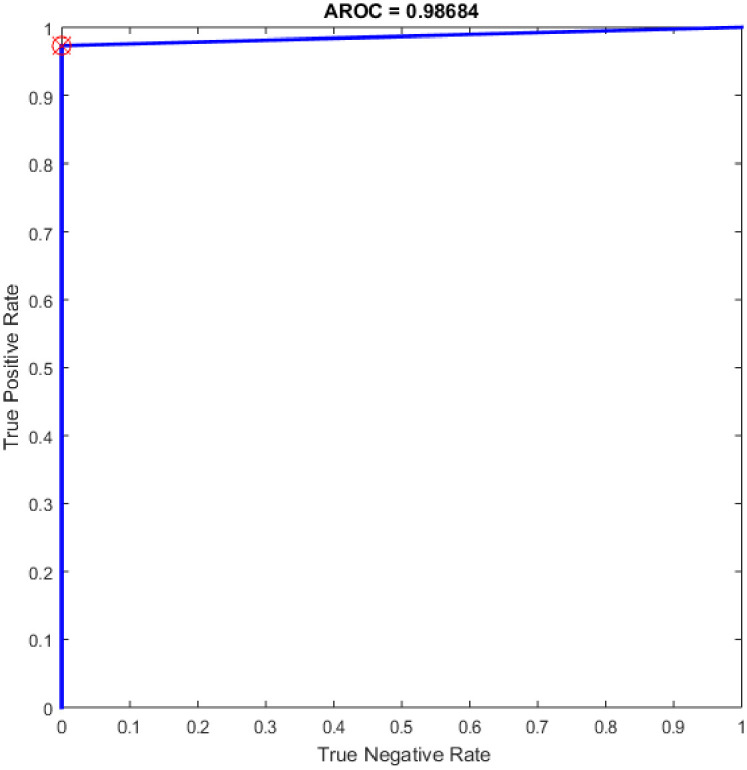
ROC of the NN for the highest result.

Additional experiments were conducted using the feedforward neural network (NN) as a classifier and HOG–PCA features. The NN was implemented in COVID-19 detection by varying the hidden neuron numbers in the range of 100–300 with a step of 25. NNs have been frequently used in a variety of applications with great success due to their ability to approximate complex nonlinear mappings directly from input patterns [41]. Namely, NNs do not require a user-specified problem-solving algorithm, but they could learn from existing examples, much like human beings. In addition, NNs have inherent generalization ability. This means that NNs could identify and synchronously respond to the patterns that are similar with but not identical to the ones that are employed to train NNs. It worth mention that the NN classifier has reimplemented for comparison purpose with the proposed OGA-ELM classifier. More details about NN can find in [42, 43]. Table 6 presents the evaluation results of the NN through in all experiments. Additionally, ROC analysis of the NN for the highest result is presented in Fig 18.

**Table 6:** Evaluation results based on NN.

Number of Hidden Neurons	tp	tn	fp	fn	Accuracy	Precision	Recall	F-measure	G-mean
**100**	36	38	0	2	97.37	100.00	94.74	97.30	97.33
**125**	36	38	0	2	97.37	100.00	94.74	97.30	97.33
**150**	37	38	0	1	98.68	100.00	97.37	98.67	98.68
**175**	37	38	0	1	98.68	100.00	97.37	98.67	98.68
**200**	36	38	0	2	97.37	100.00	94.74	97.30	97.33
**225**	37	38	0	1	98.68	100.00	97.37	98.67	98.68
**250**	36	38	0	2	97.37	100.00	94.74	97.30	97.33
**275**	36	37	1	2	96.05	97.30	94.74	96.00	96.01
**300**	36	38	0	2	97.37	100.00	94.74	97.30	97.33

The NN is regarded as a state-of-the-art technique, and many researchers have used it in health care domains, including COVID-19 detection using chest X-ray images [8, 44–47]. Therefore, this study compared the proposed approaches of OGA–ELM (random, K-tournament, and roulette wheel) with the NN approach to evaluate the performance of OGA–ELM (random, K-tournament, and roulette wheel). As shown in the experimental results in Tables 3–6, OGA–ELM (random, K-tournament, and roulette wheel) outperforms the NN in all experiments. The accuracy of OGA–ELM (random, K-tournament, and roulette wheel) with 100–300 hidden neurons is higher than that of the NN. This finding indicates that the performance results of OGA–ELM (random, K-tournament, and roulette wheel) are better than those of NN in all iterations. Tables 3–6 demonstrate the comparative results between the NN and OGA–ELM (random, K-tournament, and roulette wheel) in terms of false negative, true negative, false positive, true positive, recall, accuracy, G-mean, precision, and F-measure for all the conducted experiments. The highest accuracy was obtained by OGA-ELM (roulette wheel) with (150, 200–300) neurons, followed by OGA-ELM (K-tournament) with (225–300) neurons, OGA-ELM (random) with (150, 275, and 300) neurons, and the NN with (150, 175, and 225) neurons, as shown in Tables 3–6. The achieved accuracies were 100.00% for OGA–ELM (random, K-tournament, and roulette wheel) and 98.68% for NN. The other measures results for the NN were as follows: precision (100.00%), recall (97.37%), F-measure (98.67%), and G-mean (98.68%). The results for OGA–ELM (random, K-tournament, and roulette wheel) were as follows: precision (100.00%), recall (100.00%), F-measure (100.00%), and G-mean (100.00%).

**Fig 19:**
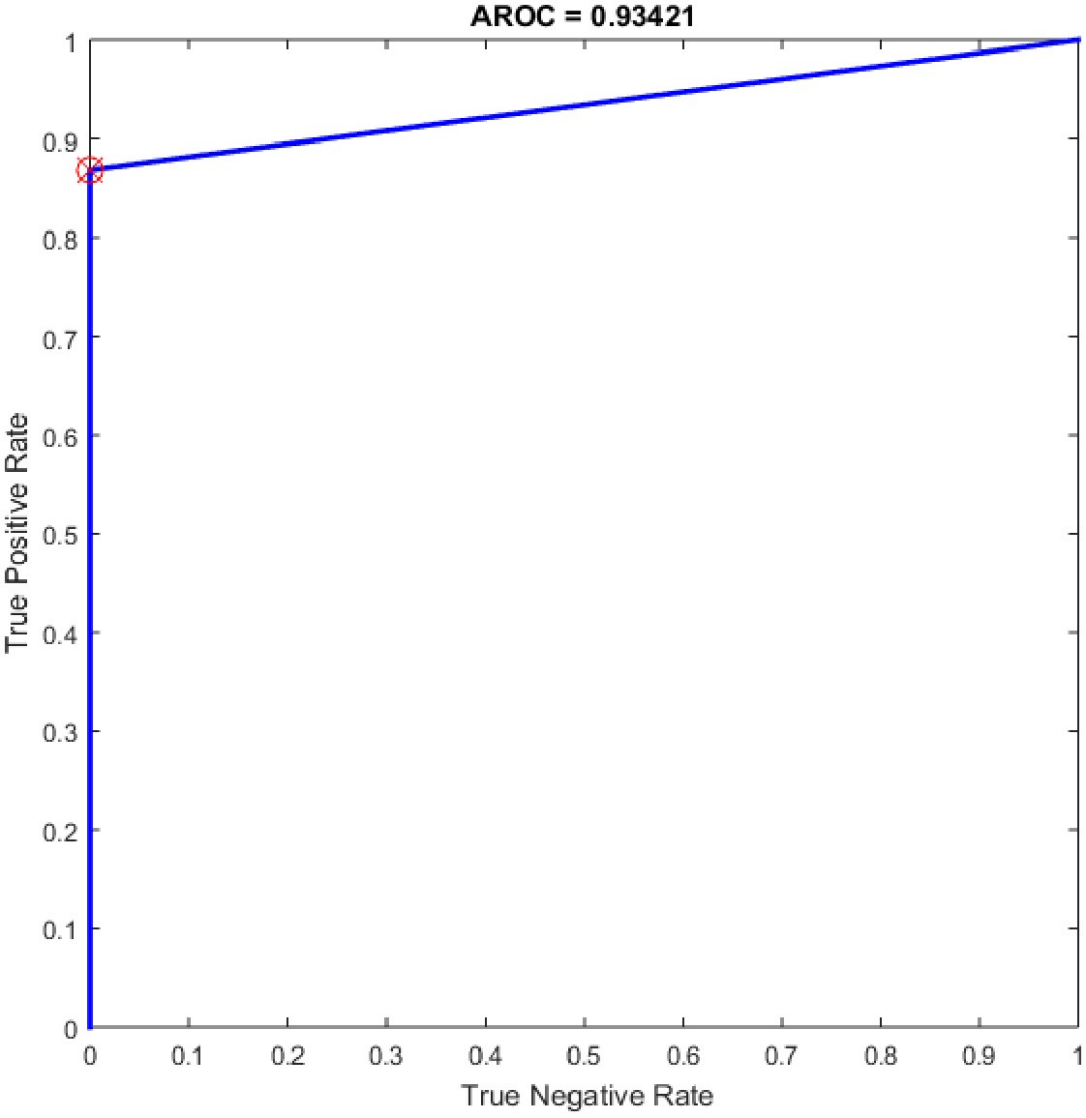
ROC of the ELM for the highest result.

**Fig 20:**
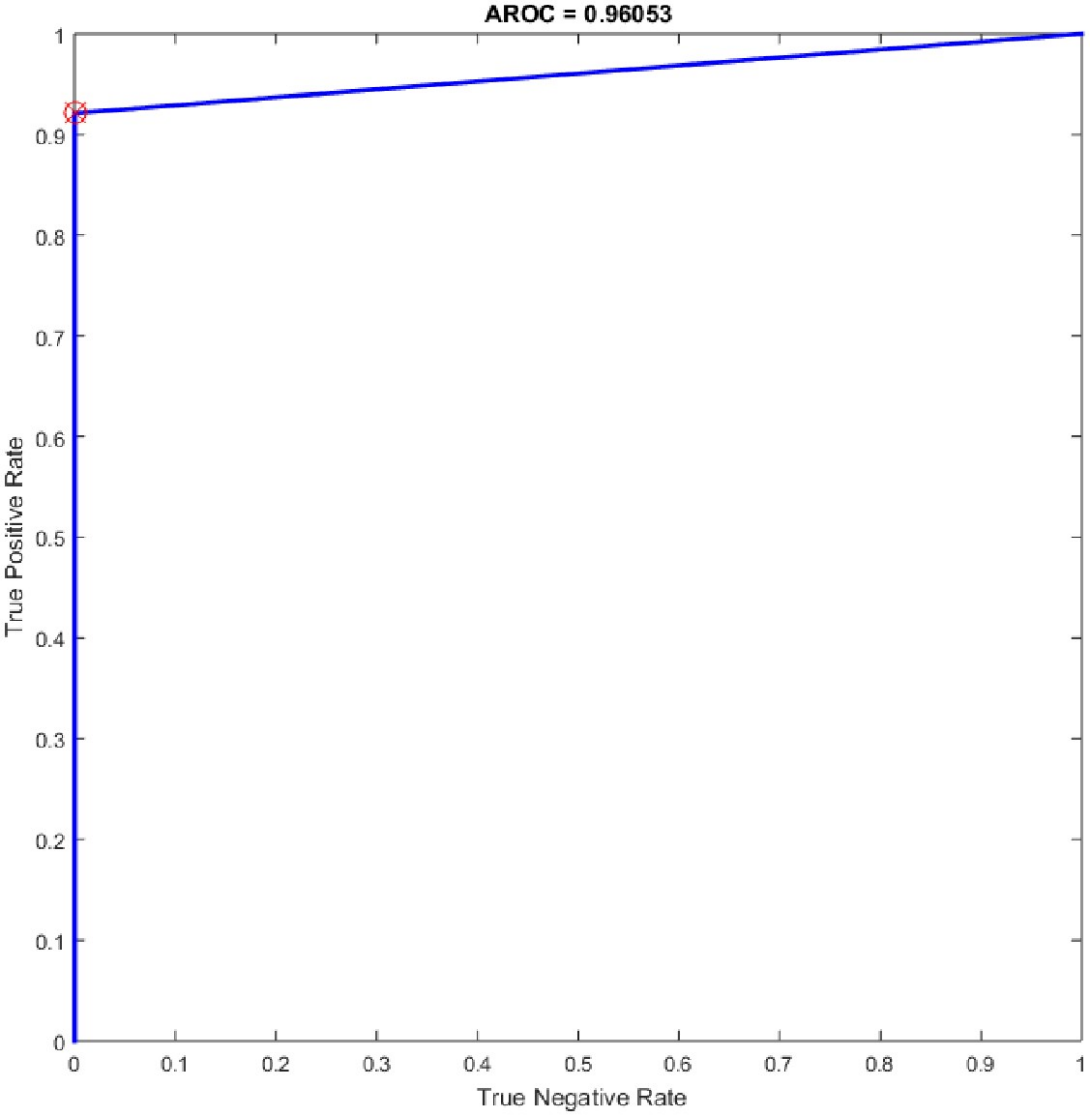
ROC of the FLN for the highest result.

Several experiments were performed for the basic ELM and fast learning network (FLN) with varying numbers of hidden neurons within the range of 100–300 with an increment of 25. ELM is a novel single hidden layer feedforward neural network (SLFN) where the input weights and the bias of hidden nodes are generated randomly without tuning and the output weights are determined analytically. While the FLN is based on the thought of ELM [19]. In FLN, the input weights and hidden layer biases are randomly generated, and the weight values of the connection between the output layer and the input layer and the weight values connecting the output node and the input nodes are analytically determined based on least-squares methods [48]. It worth mention that the FLN classifier has reimplemented for comparison purpose with the proposed OGA-ELM classifier. More details about FLN can find in [48]. Tables 7 and 8 provide the experiment results of the basic ELM and FLN. The highest performance of the basic ELM was achieved with 250 neurons, and the achieved accuracy was 93.42%. The results of other evaluation measures were 92.96%, 100.00%, 86.84%, and 93.19% for F-measure, precision, recall, and G-mean, respectively. The highest performance of the FLN was achieved with 275 and 300 neurons, and the achieved accuracy was 96.05%. The results of other evaluation measures were 95.89%, 100.00%, 92.11%, and 95.97% for F-measure, precision, recall, and G-mean, respectively. Figs 19 and 20 are show the ROC of the basic ELM and FLN for the highest obtained results.

**Table 7:** Evaluation results based on basic ELM.

Number of Hidden Neurons	tp	tn	fp	fn	Accuracy	Precision	Recall	F-measure	G-mean
**100**	28	32	6	10	78.95	82.35	73.68	77.78	77.90
**125**	27	30	8	11	75.00	77.14	71.05	73.97	74.04
**150**	33	30	8	5	82.89	80.49	86.84	83.54	83.60
**175**	27	35	3	11	81.58	90.00	71.05	79.41	79.97
**200**	31	31	7	7	81.58	81.58	81.58	81.58	81.58
**225**	31	33	5	7	84.21	86.11	81.58	83.78	83.81
**250**	33	38	0	5	93.42	100.00	86.84	92.96	93.19
**275**	33	36	2	5	90.79	94.29	86.84	90.41	90.49
**300**	32	36	2	6	89.47	94.12	84.21	88.89	89.03

**Table 8:** Evaluation results based on FLN.

Number of Hidden Neurons	tp	tn	fp	fn	Accuracy	Precision	Recall	F-measure	G-mean
**100**	34	38	0	4	94.74	100.00	89.47	94.44	94.59
**125**	33	38	0	5	93.42	100.00	86.84	92.96	93.19
**150**	33	38	0	5	93.42	100.00	86.84	92.96	93.19
**175**	32	38	0	6	92.11	100.00	84.21	91.43	91.77
**200**	34	38	0	4	94.74	100.00	89.47	94.44	94.59
**225**	33	38	0	5	93.42	100.00	86.84	92.96	93.19
**250**	34	38	0	4	94.74	100.00	89.47	94.44	94.59
**275**	35	38	0	3	96.05	100.00	92.11	95.89	95.97
**300**	35	38	0	3	96.05	100.00	92.11	95.89	95.97

**Fig 21:**
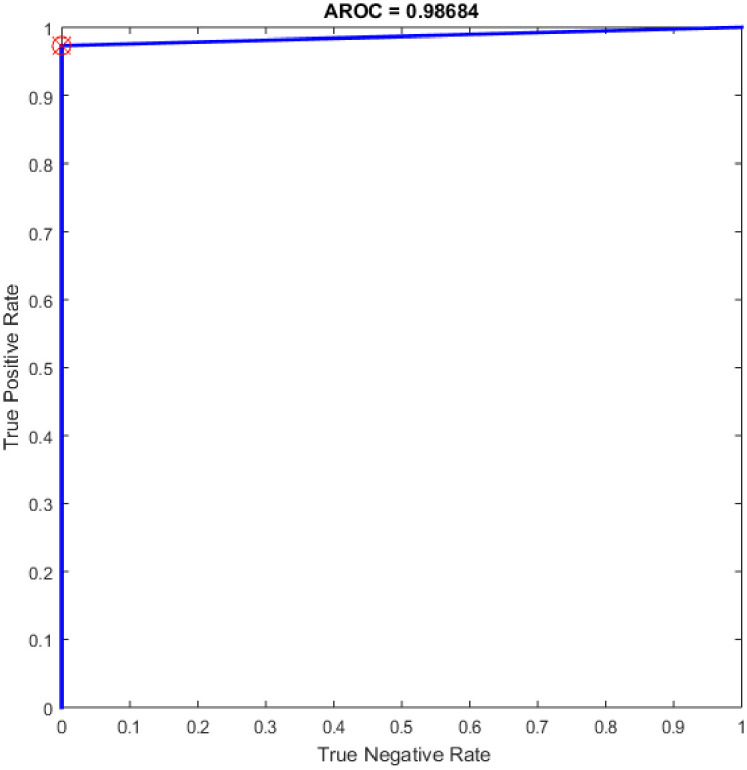
ROC of the SVM for the highest result.

Additional experiments were conducted using SVM (linear kernel) and SVM (precomputed kernel). The term of SVM was first suggested in [49] on the foundation of statistical learning theory. It has turned into the main part of machine learning methods. It was created for binary sorting (classification). The main advantage of SVM classifier is to discover the improved decision border that exemplifies the greatest decisiveness (maximum margin) amidst the classes. The standard of SVM begins from resolving the problems of linear separable then expands to treat the non-linear cases. SVM develops a hyperplane that isolates two classes and attempts to accomplish utmost separation between the classes [50]. It worth mention that the SVM classifier has reimplemented for comparison purpose with the proposed OGA-ELM classifier. More details about SVM can find in [51, 52]. Table 9 provides the experiment results of SVM (linear kernel) and SVM (precomputed kernel). Fig 21 is show the ROC of the SVM for the highest obtained result.

**Table 9:** Evaluation results based on SVM.

	tp	tn	fp	fn	Accuracy	Precision	Recall	F-measure	G-mean
**SVM (linear kernel)**	31	38	0	7	90.79	100.00	81.58	89.86	90.32
**SVM (precomputed kernel)**	35	38	0	3	96.05	100.00	92.11	95.89	95.97

**Fig 22:**
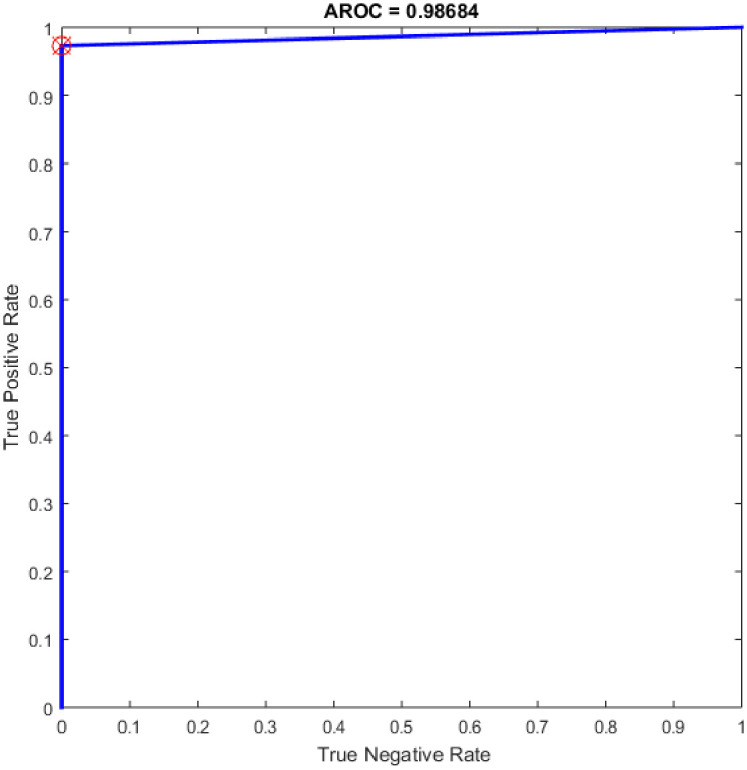
ROC of the CNN for the highest result.

Furthermore, additional experiments have been conducted based on CNN in COVID-19 detection using the same dataset (see section 3.1). CNN architectures consist of two bases namely convolutional base and classifier base. The convolutional base includes three major types of layers are: a convolutional layer, an activation layer, and a pooling layer, utilized to discover the critical features of the input images, called feature maps. While the classifier base includes the dense layers that convert the feature maps to one dimension vectors to expedite the classification task using a number of neurons [53]. It worth mention that the CNN algorithm has reimplemented for comparison purpose with the proposed OGA-ELM classifier. More details about CNN can find in [54, 55]. Table 10 illustrates the CNN architecture, while Table 11 depicts the hyper-parameters of the model. The highest performance of the CNN was achieved an accuracy of 96.05%. While the results of other evaluation measures were 96.10%, 94.87%, 97.37%, and 96.11% for F-measure, precision, recall, and G-mean, respectively. The ROC of CNN for the highest result is show in Fig 22.

**Table 10:** The CNN architecture factors.

Layer Name	CNN
Input Image	128x128x1 images with ‘zerocenter’ normalization
Convolution	8 3x3 convolutions with stride [1 1] and padding ‘same'
Batch Normalisation	Batch normalisation
Activation Function	ReLU
Max Pooling	2x2 max pooling with stride [2 2] and padding [0 0 0 0]
Convolution	16 3x3 convolutions with stride [1 1] and padding ‘same'
Batch Normalisation	Batch normalisation
Activation Function	ReLU
Max Pooling	2x2 max pooling with stride [2 2] and padding [0 0 0 0]
Convolution	32 3x3 convolutions with stride [1 1] and padding ‘same'
Batch Normalisation	Batch normalisation
Activation Function	ReLU
Fully Connected	2 fully connected layer
Softmax	softmax
Output Classification	crossentropyex

**Table 11:** The trained model parameters used in COVID-19 detection.

Hyper-Parameters	Values
Optimisation Method	SGDM
Rate of Learning	0.01
Max Epochs	4
Shuffle	every-epoch
Frequency Validation	30
Momentum	0.90
Batch Size	128

**Fig 23:**
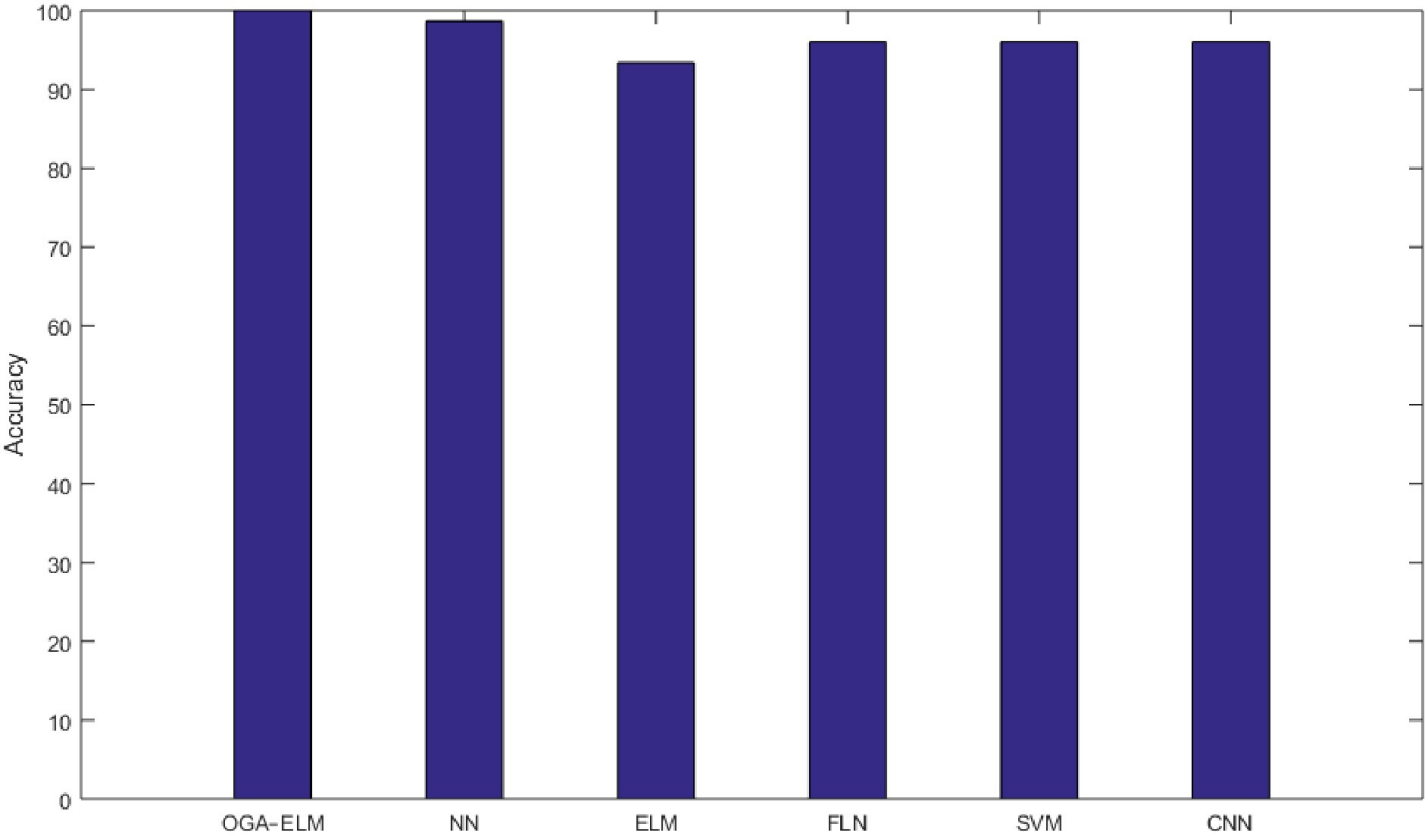
The highest achieved accuracy for all methods.

As the results shown in Tables 3–9 and 12, the performance of OGA–ELM (random, K-tournament, and roulette wheel) outperformed the NN, basic ELM, FLN, SVM, and CNN in all experiments. Therefore, the performance of OGA–ELM (random, K-tournament, and roulette wheel) was very impressive, with an accuracy of 100.00%. Besides, Fig 23 shows the comparison of the highest achieved accuracies for OGA-ELM, NN, basic ELM, FLN, SVM, and CNN.

**Table 12:** Evaluation results based on CNN.

	tp	tn	fp	fn	Accuracy	Precision	Recall	F-measure	G-mean
**CNN**	37	36	2	1	96.05	94.87	97.37	96.10	96.11

In addition, the proposed method has fast computation time in all experiments with only a few seconds for detection. This study confirms the combination of the HOG-PCA features with OGA–ELM classifier is an efficient system for COVID-19 detection using chest X-ray images that could help doctors in easily detecting COVID-19 in clinical practice. Furthermore, in order to evaluate the proposed OGA-ELM in the detection of COVID-19, Table 13 shows the comparison of accuracy between our method with other recent methods in [9, 56–60] which are worked on the detection of COVID-19 using deep learning and machine learning algorithms.

**Table 13:** Comparison of accuracies between methods.

Methods	Accuracy
Our Method (OGA-ELM)	100.00%
Method in [58]	97.48%
Method in [56]	95.12%
Method in [57]	98%
Method in [60]	94.1%
Method in [9]	95.38%
Method in [59]	90%

Based on Table 13, it shows that the proposed OGA-ELM method has outperformed all methods in terms of accuracy for COVID-19 detection. However, this work has some limitations that can be summarized as follow:

- The images dataset that used for training and testing are small.
- The proposed method has focused on classifying images into two classes only, healthy or COVID-19, and ignoring other lung diseases.

## 4. Conclusion

We have proposed the histogram oriented gradient-principal component analysis (HOG-PCA) features and optimised genetic algorithm-extreme learning machine (OGA-ELM) (with random, K-tournament, and roulette wheel selection mechanism) approaches using chest X-ray images, to detect COVID-19 disease efficiently. We used a benchmark dataset of chest X-ray images that were collected from COVID-19 patients and healthy people to evaluate the efficacy of the proposed method. Results showed that the OGA–ELM (random, K-tournament, and roulette wheel) exhibit remarkable performance and achieves 100.00% accuracy. In addition, no machine learning was expected to perform 100% accurately but only be achieved by managing data. This demonstrated that the OGA-ELM had improved the effectiveness (accuracy) of the automatic COVID-19 detection compared to neural network (NN), basic extreme learning machine (ELM), fast learning network (FLN), support vector machine (SVM), and convolutional neural network (CNN). Indeed, the HOG-PCA features with low dimensionality had enhanced the efficiency (computational time), and required less memory space, where the low dimensionally lead to speed up the classification process and requires low memory space. This work provides insights into the application of HOG–PCA features with OGA–ELM (random, K-tournament, and roulette wheel) to detect COVID-19 in early stage. In future research, the classification performance of the OGA–ELM (random, K-tournament, and roulette wheel) models based on HOG–PCA features can be tested on a dataset with a high number of images. In addition, another future research can include using the OGA-ELM in other healthcare applications.
